# Effect of the Digital Education Package (DEP) on prevention of anxiety in hospitalized children: a quasi-experimental study

**DOI:** 10.1186/s12912-022-01113-8

**Published:** 2022-11-25

**Authors:** Masoumeh Zamani, Abdolhosein Emami Sigaroudi, Moluk Pouralizadeh, Ehsan Kazemnejad-Leili

**Affiliations:** 1 Department of Nursing, Shahid Beheshti School of Nursing and Midwifery, Guilan University of Medical Sciences, Rasht, 4146939841 Iran; 2 Cardiovascular Diseases Research Center, Department of Cardiology, Heshmat Hospital, School of Medicine, Guilan University of Medical Sciences, Rasht, Iran; 3 Department of Biostatistics, Shahid Beheshti School of Nursing and Midwifery, Guilan University of Medical Sciences, Rasht, Iran

**Keywords:** Hospital education, Anxiety, Child, Digital, Hospitalization

## Abstract

**Background:**

Hospitalization of children is a stressful event. However, the child’s education at the time of hospital admission can be effective for the prevention of their anxiety via the use of more attractive methods. The study’s aim was to assess the effectiveness of the education using a digital education package on the level of anxiety of hospitalized children.

**Methods:**

This is a quasi-experimental study with the randomized block method. The sample size was calculated based on Shahrabadi et al.’s study and sixty eligible hospitalized children were allocated to the two study groups from June 2019 to December 2020, in Hefdah-e-Sahrivar hospital which is the central pediatric hospital in Rasht city. The intervention was education using a digital package that was done 15 min after the hospitalization of the children. Pediatrics’ Spielberger’s anxiety Inventory was used for measuring the participants’ anxiety before and after the intervention. We used Chi square test, Fisher exact test and paired t-test to analyze data. A *p*-value < 0.05 was considered statistically significant.

**Results:**

In the post-intervention phase, total mean scores of anxiety were significantly lower in the experimental group (60.17 ± 6.46) rather than in the control group (72.6 ± 8.83) (*P* < 0.001). The mean anxiety scores before and after the intervention were 87.43 ± 11 vs. 60.17 ± 6.46 in the intervention group and 81.5 ± 11 vs. 72.6 ± 8.83 in the control group, respectively. There were significant differences in intergroup anxiety scores between the two study groups (*P* < 0.001).

**Conclusions:**

The current study showed that the Digital Education Package (DEP) is an effective method for reducing children’s anxiety during hospitalization. Therefore, we recommended it as a preferred and simple method rather than routine education for pediatric nurses.

## Introduction

Hospitalization is a stressful event and a source of anxiety for both the children and their parents [1]. Thirty percent of children at least one time and about 5% of them several times are hospitalized during their childhood [2]. It is a threatening and harmful experience to the physiological and psychological health of children [3]. More than 80% of children during their hospitalization receive different invasive procedures. The hospital environment and the care-related procedures such as venipuncture, blood sampling, and other therapeutic interventions are the stressors for hospitalized children [4, 5].

As the hospitalized children are unaware of the reason for their hospitalization and the invasive procedures, it can lead to an undesirable experience and psychological symptoms such as anger, uncertainty, anxiety, and feelings of helplessness [6]. The sense of helplessness associated with anxiety leads to feelings of powerlessness in children. Excessive anxiety can increase children’s negative emotions and decrease their sense of control and compatibility with medical treatment. Also, lack of cooperation, post-traumatic stress disorder, regression in behaviors, and sleep disruption are the common outcomes of children’s anxiety [7, 8].

According to the studies, although older children are able to understand the disease and hospitalization, they are still vulnerable to events such as hospitalization that decrease their sense of power [6, 8]. Pediatric nurses should establish a suitable interaction with individuals of all ages and socioeconomic classes [9]. They play key roles in supporting and controlling the negative emotions of children. Therefore, nurses should apply methods that reduce feelings such as anxiety in hospitalized children. It is necessary to develop effective strategies to reduce anxiety in children experiencing medical situations [10]. Literatures show that children who participate in educational programs are better able to control anxiety and therefore experience lower levels of preoperative anxiety [11–13]. Patients’ education using techniques that engage patients is an attractive way to reduce anxiety in children and their parents. However, due to the high workload of nursing staff and the organizational limits, the education of children in the hospital may be difficult [11, 14]. Among the approaches for patients’ education in hospitals, Digital Education Package (DEP) is an extremely popular and easy method that can facilitate the education process. Moreover, as digital education can be implemented using personal electronic devices such as tablets and smartphones, it provides social distance in the COVID-19 pandemic which is an unavoidable need [15]. Several studies showed education and distraction methods can reduce the anxiety of hospitalization in children and parents [9, 10, 12, 13]. However, to the best of our knowledge, this was the first study that investigated the effect of electronic education on anxiety induced by the hospitalization of children in Iran. This study was conducted to answer the question of whether training with DEP can reduce the anxiety of hospitalized children better than the routine methods based on the cultural context and clinical environments of hospitals in Iran.

The purpose of this study was to assess the effectiveness of education using a DEP on the level of anxiety of hospitalized children.

## Materials and methods

### Research design and setting

A quasi-experimental study was conducted in the Hefdah-e-Shahrivar hospital which is a central pediatric hospital in Rasht, Iran. The participants were assigned to two groups, experimental and control.

### Participants and sampling

Based on the findings of the study of Shahrabadi et al. [16] and with a confidence interval of 95%, an error of 5%, a test power of 90%, *d* = 0.9, σ^2^ = 1.9, and with 10% attrition rate, the sample size was calculated according to the following formula as 30 patients in each group.

$$N\kern0.5em =\kern0.5em {\sigma}^2\frac{{\left({Z}_{1-a/2}\kern0.5em +\kern0.5em {Z}_{1-B}\right)}^2}{d^2}\kern0.5em +\kern0.5em \frac{{\left({Z}_{1-a/2}\right)}^2}{2}=\kern0.5em 1.9\kern0.5em \frac{{\left(1.96\kern0.5em +\kern0.5em 1.28\right)}^2}{0.9^2}\kern0.5em +\kern0.5em \frac{(1.96)^2}{2}=27$$

The participants were the hospitalized children in the age group of 9–12 years who had been admitted to the pediatric hospital due to chronic diseases and were eligible based on the inclusion criteria of the study. The inclusion criteria were: the ability to read and write, hospitalization for the first time, and not taking anti-anxiety drugs. Also, the children with cognitive and learning difficulties did not enter the study.

Sixty eligible children were entered into the study by convenience sampling method. Then, the children were randomly allocated to the intervention or control groups using numbered envelopes. Each envelope included one of two numbers, 1 for the control, or 2 for the intervention group.

### Data collection

The data collection was done from June 24, 2019, to December 24, 2020. For data collection, after obtaining the necessary permissions and the code of ethics (IR.GUMS.REC.1398.239), the investigator explained the purpose of conducting the study and, after obtaining the written consent, the demographic data were collected from the parents and the children.

The study tool was a two-section questionnaire. The first section included the demographic characteristics of the participants consisted of the child’s characteristics including age, birth rank, education level, type of disease, place of residence, and the family’s characteristics including the number of family members, history of hospitalization in each member of the family, the parent’s educational level, parent’s job and, their income. These variables were extracted from the literature review [7, 8, 17].

The second section of the study instrument was Spielberger’s State-Trait Anxiety Inventory for children (STAIC). It is a widely used assessment tool as it is easy for children to understand, and is a valid instrument for assessing pediatrics’ anxiety [18]. The pediatrics STAI is a 40-item self-report instrument that is widely used to measure anxiety symptoms in children aged 9–12 years. The first 20 items are related to the “state anxiety” (child’s feelings during responding) and the second 20 items cover the “trait anxiety” (child’s general feelings). All statements start with the stem “I feel” and the respondents have to choose among three responses, the one that best describes their state is scored on a three-point Likert scale from 1 to 3 (e.g., very comfortable, comfortable, or not comfortable). The statements of the trait anxiety scale were scored on a 3-point scale including hardly, sometimes, and never. The anxiety score was calculated based on the mean score, and the higher scores indicated higher anxiety. The score range of the total score of STAIC was from 40 to 120.

The STAIC questionnaire validity and reliability were obtained in the original study. The alpha reliability of the STAIC was 0.87. The concurrent validity of the STAIC was confirmed by its correlation with the two most widely used measures of anxiety in children including the Children’s Manifest Anxiety Scale (CMAS) [19] and the General Anxiety Scale for Children (GASC) [20]. The STAIC Anxiety scale correlated 0.75 with the CMAS and 0.63 with the GASC [21, 22]. Also, the obtained Cronbach’s alpha coefficient in the current study was 0.896.

The intervention in the current study was the education of hospitalized children using the DEP. DEP was a researcher-made educational package including electronic text, images, animations, video, and audio files to introduce the hospital healthcare team (the head nurse, nursing staff, physicians), the time of physicians’ visit, and the nursing interventions. The images were used to show the different parts of the hospital environment (such as the pediatric ward and the rooms, beds, nursing station, and the hospital para-clinical departments). In preparing the DEP, attempts were made to use understandable content for children aged 9–12 years. To design the content, framework, and function of the DEP, we consulted with an expert team that included a child psychologist, hospital nursing manager, pediatric department supervisor, and software engineer. For children in the control group, conventional patients’ education was performed with the usual simple verbal instructions and using an educational booklet. The contents in the booklet were the same as the digital content.

### Data analysis

We used SPSS software version 20.0 for the data analysis. Descriptive statistics were used to calculate the means, standard deviations, and the range of scores. According to the Shapiro-Wilk test, the anxiety scores had a normal distribution, therefore, the parametric tests have been used to analyze the scores. To examine the homogeneity of the two groups, a χ2 test or Fisher exact test was used to analyze categorical variables. Differences in the mean scores on the children’s anxiety levels between the two groups were investigated by an independent t-test. A paired t-test was performed to compare the mean scores of the inter-groups. A *p*-value < 0.05 was considered statistically significant.

### Procedure

**Fig. 1:**
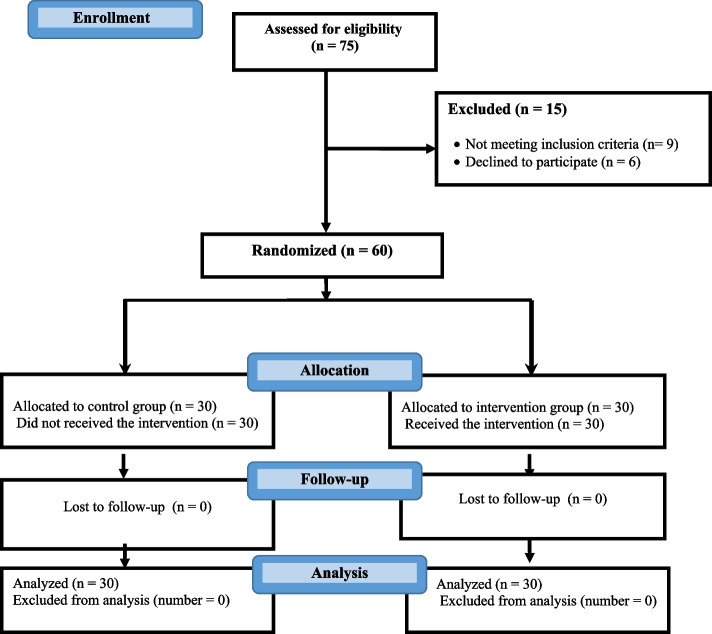
CONSORT flow diagram

Fifteen minutes after the admission to the hospital, and before each invasive procedure, the baseline anxiety score of the participated children was recorded based on STAI. Then, the researcher met the participants and their parents in the experimental group in a comfortable private room where they could watch the DEP using a laptop. During the intervention, the children were engaged with the DEP. The intervention was done during a 15 minute session. At the end of the intervention, the children completed the study questionnaire again. For the control group, no intervention was done and they received only the routine admission verbal information at their bedside using the educational pamphlet by the nursing staff. They also completed the questionnaire at similar times to the intervention group. There was no attrition in the experimental and control group (Fig. 1).

## Results

Table 1 shows the demographic characteristics of the participants. The majority of the children were the first child (60%), nine years old (45%), in the third grade (45%), and residents in the urban areas (80%). Sixty percent of the mothers and 53.33% of the fathers had a diploma degree. According to the chi-square and Fisher exact test, there was no significant difference in demographic variables scores between the study and control groups (*P* > 0.05), therefore, the two groups can be considered to be homogenous with regard to all the considered variables (Table 1).

**Table 1:** Demographic characteristics of the hospitalized children (*n* = 60)

Demographic Variables	Study Groups			
	Intervention	Control	Total	*P* -Value
	n (%)	n (%)	n (%)	
**Age (Year)**				
**9**	13(43.33)	14(46.67)	27(45)	0.662*
**10**	6(20)	4(13.33)	10(16.67)	
**11**	4(13.33)	7(23.33)	11(18.33)	
**12**	7(23.33)	5(16.67)	12(20)	
**Sex**				
Girl	19(63.33)	15(50)	34(56.67)	0.297*
Boy	11(36.67)	15(50	26(43.33)	
**Birth Rank**				
**1**	18(60)	18(60)	36(60)	0.999**
**2**	10(33.33)	9(30)	19(31.67)	
**3**	2(6.67)	3(10)	5(8.33)	
**Number of family member**				
** 3**	10(33.33)	10(33.33)	20(33.33)	
** 4**	17(56.67)	16(53.33)	33(55)	
** 5**	3(10)	4(13.33)	7(11.67)	
**Child’s education class**				
Third	13(43.33)	14(46.67)	27(45)	0.662*
Forth	6(20)	4(13.33)	10(16.67)	
Fifth	4(13.33)	7(23.33)	11(18.33)	
Sixth	7(23.33)	5(16.67)	12(20)	
**Type of disease**				
Gastrointestinal	16(53.33)	16(53.33)	32(53.33)	0.999**
Renal	9(30.)	10(33.33)	19(31.67)	
Respiratory	2(6.67)	2(6.67)	4(6.67)	
Dermal	3(10)	2 (6.67)	5(8.33)	
**Place of residence**				
Urban	25(83.33)	23(76.70)	48(80)	0.682*
Rural	5(16.67)	7(23.30)	12(20)	
**Father’s education level**				
Illiterate	0(0)	1(3.33)	1(1.67)	0.462**
Under diploma	4(13.33)	6(20)	10(16.67)	
Diploma	15(50)	17(56.67)	32(53.33)	
Academic education	11(36.67)	6(20)	17(28.33)	
**Mother’s education level**				
Illiterate	0 (0)	0 (0)	0 (0)	0.999**
Primary school	5(16.67)	7(23.33)	12(20)	
Diploma	19(63.33)	17(56.67)	36(60)	
Academic education	6(20)	6(20)	12(20)	
**Father’s job title**				
12(20)	8(26.67)	4(13.33)	12(20)	0.359*
10(16.67)	6(20)	4(13.33)	10(16.67)	
26(43.33)	12(40)	14(46.67)	26(43.33)	
12(20)	4(13.33)	8(26.67)	12(20)	
**Mother’s job title**				
Housekeeper	26(86.67)	26(86.67)	52(86.67)	0.999**
Employee	4(13.33)	4(13.33)	8(13.33)	
**Monthly family’s income (Rials)**				
< 15,000,000	5(16.67)	6(20)	11(18.33)	0.798*
15,000,000–25,000,000	18(60) 7 (23.33)	19(63.33) 5(16.67)	37(61.67) 12(20)	
> 25,000,000				
**History of hospitalization in family members**				
Yes	13(43.33)	14(46.67)	27(45)	0.795*
No	17(56.67)	16(53.33)	33(55)	

*Chi- square 2 test

**Fisher exact test

The mean anxiety scores of the children in the experimental and control groups are shown in Table 2. The independent t-test showed no significant difference in the STAIC mean scores between the experimental and control groups in the baseline(*P*>0.05).

**Table 2:** Comparison of the changes of mean scores of anxiety of children between the intervention and control groups in the baseline and the post- intervention

Variables		Study groups n(%)		*P*-value
		Control (n = 30)	Intervention(*n* = 30)	
**State anxiety**	Baseline	45.43 (6.04)	42.90 (5.17)	0.086*
	Post- intervention	29.50 (4.26)	37.73(4.66)	0.001*
	***P*-value**	0.001**	0.001**	
**Trait anxiety**	Baseline	42(6.38)	38.60 (7.01)	0.054*
	Post- intervention	30.67 (3.50)	34.87 (5.76)	0.001*
	***P*-value**	0.001**	0.001**	
**Total score**	Baseline	87.43 (11.10)	81.50 (11)	0.052*
	Post- intervention	60.17 (6.46)	72.60 (8.83)	0.001*
	***P*-value**	0.001**	0.001**	

*Independent *t*- test

**Paired *t*- test

However, in the post-intervention phase, mean scores of total anxiety, and the anxiety of state and trait were significantly lower in the experimental group compared to the control (*P* < 0.001) (Table 2).

Paired t-test showed significant differences in intergroup STAIC mean scores in the intervention group (*P* < 0.001) and control group (*P* < 0.001), in the two stages before and after the intervention (Table 2).

In the post-intervention phase, total mean scores of anxiety were significantly lower in the experimental group (60.17 ± 6.46) rather than in the control group (72.6 ± 8.83) (*P* < 0.001). The mean anxiety scores before and after the intervention were 87.43 ± 11 vs. 60.17 ± 6.46 in the intervention group and 81.5 ± 11 vs. 72.6 ± 8.83 in the control group, respectively. There were significant differences in intergroup anxiety scores between the two study groups (*P* < 0.001).

## Discussion

This study evaluated the effect of DEP on the level of anxiety in hospitalized school-age children. To our knowledge, this is the first study to show the effect of DEP on hospitalized pediatrics’ anxiety in Iran. The results of the present study showed that the levels of anxiety scores in the two control and intervention groups were significantly different after the intervention and the DEP reduced the anxiety level of the children in the total anxiety score, and both state and trait anxiety scores. In addition, assessing the intergroup differences revealed a decreased anxiety score from pre to post-intervention in each group.

The present study showed that DEP reduced children’s anxiety after admission to the hospital. Consistent with the findings, the other studies reported educational programs can effectively reduce the anxiety rate of school-aged hospitalized children [12, 13, 23]. Wantanakorn et al., showed that digital education using a mobile application can reduce the rate of anxiety in hospitalized children by providing hospital routine information [24]. In line with our results, another study that evaluated the effectiveness of an electronic education tour by a mobile application on the anxiety of the pediatrics who were candidates for the operation room showed that children in the intervention group had significantly lower mean scores of anxiety [11]. Sadeghian et al. reported the program of preparation for hospitalization was an effective method for decreasing the anxiety of school-age children after admission to the hospital [25]. According to the psychosocial development theory of Erikson, school-aged children have a sense of superiority. In these children, the occurrence of situations that might lead to a sense of inferiority and loss of control such as illness and the anxiety of hospitalization may be harmful [17]. The interventions that have a great influence on decreasing hospital anxiety and stress can improve their relaxation and decrease the duration of hospitalization in hospitalized children. The education of the hospitalized children and their parents about hospital routines and the introduction of the health provider team are some of these measures [2].

In the present study, scores of the total anxiety, and state and trait anxiety were significantly decreased from pre to post-intervention in the intervention group. The finding indicated that DEP was a more effective method for decreasing anxiety in hospitalized children in comparison to the conventional method of face-to-face education. It demonstrates the familiarization of hospitalized children with the stressful environment of the hospital using various audiovisual media such as the DEP is an effective method on control of anxiety level in children.

The results showed that in addition to the state anxiety, trait anxiety, which is the structural component of anxiety level, was affected by the hospitalization experience. DEP was a comprehensive electronic education to introduce the hospital in a short time. As digital devices are attractive for children such as play therapy, they can create a distraction that is an important factor in reducing children’s anxiety [8, 13].

Studies on the effect of electronic education on anxiety in hospitalized children are rare, however, in a study that compared the effect of an informative leaflet with verbal conventional information on reducing the hospitalized children’s anxiety, the authors reported decreased anxiety and increased satisfaction in the intervention group [26]. It is inferred that written and visual educational programs compared to verbal education can be more effective in decreasing pediatrics’ anxiety. However, in contrast with the findings, another study showed trait anxiety did not differ in the hospitalized children in the intervention group (education by a leaflet) vs. the control group (conventional education), and the level and prevalence of anxiety in the two study groups significantly increased after the intervention [27]. Delvecchio et al., also reported trait anxiety did not significantly differ between the two study groups, and the trait anxiety that is related to children’s everyday life experiences was not affected by hospitalization [8]. Differences in the children’s age, hospital environment, organizational culture, and routines might explain the inconsistency. Other studies also have reported the role of electronic education in creating relaxation and reducing anxiety and negative emotions in sick and healthy children [28–30].

In our study, regardless of the method of education and providing information to the hospitalized children, the trend of intergroup changes showed that anxiety was significantly reduced from pre to post-intervention in each group. This result demonstrated that in addition to the DEP, routine education was also effective in decreasing the anxiety of children after hospitalization, however, DEP was more effective in decreasing the anxiety. The purpose of patient education is to provide increased self-awareness and the new methods can suitably control the emotional reactions of the patients or their family members. Patient education is an important component of nursing care in pediatrics departments. Providing children with enough information about the hospital environment allows them to achieve adaptation and control over situations. Also, it reduces children’s uncertainty and anxiety [31, 32]. It seems that providing information about hospital and clinical processes through electronic education to the child and parents during the hospitalization process is a useful social support method and can create a sense of empathy for the child and parents. Düken et al. indicated healthcare professionals can contribute to reducing the anxiety and loneliness of parents of children under liver transplantation by strengthening their social support systems [10]. Furthermore, it is possible the sense of independence in the children using education through an electronic device was an important factor in the greater impact of DEP than the conventional patients’ education method.

### Implications and future research

Our findings highlight the importance of education by DEP as a facilitator for patients’ education and orientation in the pediatrics hospital. The study provides good evidence regarding the effect of DEP on the anxiety caused by hospitalization in children. The results indicate DEP actively engages hospitalized children and effectively reduces their anxiety. DEP is an easy strategy and a more effective method for reducing the anxiety of hospitalized children. Therefore, it is recommended pediatric nurses apply DEP for the prevention of anxiety in hospitalized children. Future research in the different pediatric clinical settings is recommended for the generalization of the findings.

### Limitations

This research has some limitations. As the participants were selected from children affected by different kinds of chronic diseases, therefore, it is unclear whether this method is effective in children with acute diseases. The small sample size is also another study limitation.

## Conclusion

The findings of the current study demonstrated that education by DEP was an effective method of reducing the anxiety caused by hospitalization in children. Since DEP is an easy, accessible, and independent method for educating hospitalized children and their families, it is recommended that it be used as a preferred method to conventional and routine education using paper pamphlets. In addition, this method can minimize the possibility of transmitting infectious diseases, such as COVID-19, in the hospital that today is a major crisis for mankind in the world.

## Data Availability

According to the quality improvement project intent, at the time of the decision, the local institutional boards recommended storing the database at the institutional level. Therefore, the dataset used and analyzed is available from the corresponding author on request.
